# Use and safety of prophylactic endoscopy from a single center serving urban and rural children with portal hypertension

**DOI:** 10.1038/s41598-021-03759-x

**Published:** 2022-01-07

**Authors:** Voytek Slowik, Anissa Bernardez, Heather Wasserkrug, Ryan T. Fischer, James F. Daniel, Tassos Grammatikopoulos

**Affiliations:** 1 Department of Pediatrics, University of Missouri–Kansas City School of Medicine, 2411 Holmes Street, Kansas City, MO 64108 USA; 2 Division of Gastroenterology Hepatology and Nutrition, Children’s Mercy Kansas City, 2401 Gillham Rd, Kansas City, MO 64108 USA; 3 University of Missouri–Kansas City School of Medicine, 2411 Holmes Street, Kansas City, MO 64108 USA; 4 Paediatric Liver Gastroenterology and Nutrition Centre and MowatLabs, King’s College Hospital NHS Foundation Trust, London, SE5 9RS UK

**Keywords:** Diseases, Gastroenterology, Medical research

## Abstract

Prophylactic endoscopy is routine in adults with portal hypertension (PHTN), but there is limited data in pediatrics. We sought to describe our experience with prophylactic endoscopy in pediatric PHTN. This is a retrospective study of 87 children who began surveillance endoscopy prior to gastrointestinal bleeding (primary prophylaxis) and 52 who began after an episode of bleeding (secondary prophylaxis) from 01/01/1994 to 07/01/2019. Patients who underwent primary prophylaxis had a lower mean number of endoscopies (3.897 vs 6.269, p = 0.001). The primary prophylaxis group was less likely to require a portosystemic shunt (6% vs 15%, p < 0.001) with no difference in immediate complications (1% vs 2%, p = 0.173) or 2-week complications (1% vs 2%, p = 0.097). No deaths were related to variceal bleeding or endoscopy. Kaplan–Meier Survival Curve suggests improved transplant and shunt free survival in the primary prophylaxis group (log-rank p < 0.001). Primary and secondary endoscopic prophylaxis should be considered safe for the prevention of variceal hemorrhage in pediatric portal hypertension. There are differences in outcomes in primary and secondary prophylaxis, but unclear if this is due to patient characteristics versus treatment strategy. Further study is needed to compare safety and efficacy to watchful waiting.

## Introduction

Portal hypertension (PHTN) is a well-established risk factor for gastrointestinal hemorrhage in pediatrics^1^. It is the result of obstructed blood flow through the portal venous system at the prehepatic, sinusoidal or post hepatic level. The underlying causes are variable and include portal vein thrombosis, mass effect, intrinsic liver disease and cirrhosis, among others. This increased resistance leads to an elevated portosystemic gradient (PSG) resulting in the development of varices as blood travels through shunts to bypass the obstructed portal system and return to venous circulation. While many complications can arise from PHTN, gastrointestinal hemorrhage secondary to varices requires emergent evaluation and treatment to prevent morbidity and mortality^2^.

However, the role of preventative management for esophageal varices does not have consensus between children and adults^2,3^. In adults with PHTN without an episode of bleeding, there are recommendations for primary preventative endoscopy to reduce the morbidity and mortality from variceal bleeding^2^. Endoscopy may lead to treatment decisions including use of non-selective beta blockers (NSBB), endoscopic variceal ligation (EVL), and portosystemic shunting. In pediatrics, there is a debate about the role of NSBB therapy and EVL in the prevention of bleeding complicated by a paucity of data^3^. There are multiple calls for further research to establish the safety and efficacy of prophylactic endoscopy and associated long term outcomes and create more objective guidance on treatment^4,5^. Indeed, some centers have already published data to challenge these recommendations including Duché et al.^6,7^. Additionally, Molleston et al. have recently published a 6 week mortality rate for variceal bleeding of 8.8% in the United States with Black or Hispanic children at higher risk^8^. While this rate is lower than in adults, it remains a significant risk to children and deserves further attention.

Our tertiary medical center serves a significant rural population who do not always have immediate access to appropriate medical care in the setting of variceal hemorrhage. With this population, we have historically adopted a primary prophylactic approach to endoscopy for variceal hemorrhage that has continued with some patients developing gastrointestinal hemorrhage prior to endoscopy and others more rarely choosing secondary prophylaxis. In this retrospective cohort study, our primary aim was to describe our experience and safety parameters with prophylactic endoscopy (as described in “Methods”) to prevent gastrointestinal bleeding or recurrent gastrointestinal bleeding in pediatric PHTN. We also sought to describe outcomes in portosystemic shunt and transplant free survival. Lastly, we sought to determine if there were significant differences between our primary and secondary prophylactic endoscopy groups.

## Methods

The Institutional Review Board at Children’s Mercy—Kansas City approved all research activities and conformed ethical approval to the guidelines of the 1975 Declaration of Helsinki. Given the retrospective nature of the study, a waiver of consent was approved by the same board. Subjects were retrospectively identified using an internal electronic health record system database search. Patients met inclusion criteria if they had a diagnosis of PHTN and underwent initial endoscopy prior to age 18 years. Patients were grouped into primary prophylaxis if their initial endoscopy was for variceal screening due to the clinical diagnosis of hypersplenism or other manifestation of PHTN without history of gastrointestinal bleeding or anemia. Manifestations of PHTN included thrombocytopenia, ascites, radiography demonstrating signs of PHTN or other manifestation of PHTN. The secondary prophylaxis group included those with PHTN whose initial endoscopy at our center was to manage gastrointestinal bleeding or for surveillance after an episode of bleeding initially managed at an outside center. Some were from families who purposefully deferred endoscopy while the majority were those who developed bleeding prior to initial endoscopy. Hemoptysis and anemia (as indications for endoscopy) were also considered to be symptoms of gastrointestinal bleeding to avoid undercounting bleeding episodes. Patients who had PHTN but did not undergo endoscopy were not included due to insufficient numbers for analysis. Clinical data were entered into a secured Research Electronic Data Capture database for further analysis. Data included age at time of endoscopy, underlying diagnosis, indication for endoscopy, endoscopic reports, and relevant clinical outcomes from January 01, 1994 to July 01, 2019. Pediatric End-Stage Liver Disease (PELD) and Model for End-Stage Liver Disease (MELD) scores were collected if available within 2 months of initial endoscopy excluding patients with cavernous transformation of the portal vein or portal vein thrombosis. If present, varices were described as grades 1–3 based on currently accepted standards^2,7^.

### Institutional protocol

Patients with PHTN are clinically diagnosed at the Liver Care Center at Children’s Mercy–Kansas City, using a combination of history, physical examination, laboratory, and radiographic findings by one of three board certified pediatric transplant hepatologists in patients with an underlying diagnosis that could cause PHTN. The PSG is not routinely measured in our practice as its utilization has not been validated in children. Clinical features of portal hypertension placing a patient at risk for varices include physical exam findings such as telangiactasias and palmar erythema, splenomegaly on exam or imaging, ascites on exam or imaging, thrombocytopenia, varices on imaging, hepatofugal blood flow on Doppler ultrasound, among others. If PHTN is clinically suspected, patients are offered endoscopy for primary prophylaxis prior to an episode of variceal bleeding. At endoscopy, EVL is the treatment of choice for large esophageal varices, red wale signs, or with active hemorrhage for those where the banding apparatus can fit, typically after age 4 years or weight greater than 10 kg. Sclerotherapy is used if EVL is not feasible in the setting of active hemorrhage due to patient size, but is not routinely used in primary prophylaxis. Follow up endoscopy is repeated until varices are obliterated at an interval determined by the primary hepatologist based on presentation and severity, typically 2 weeks to 3 months. Afterwards, patients undergo surveillance endoscopy every 1–3 years with interval dependant on severity of varices, history of bleeding, progression of liver disease, and ability of family to travel to hospital. Non-selective beta blockers in the form of propanolol are started in those who have breakthrough bleeding despite prophylactic endoscopy at the descretion of the primary hepatologist. Those with refractory varices or recurrent bleeding are referred for portosystemic shunting or liver transplantation (LT). Patients who present initially with variceal bleeding receive a similar secondary surveillance endoscopic schedule after control of bleeding and obliteration of varices. They are started on propranolol and referred for portosystemic shunt or LT for the same indications.

### Statistical analysis

Patient and endoscopy characteristics were compared between the groups using t-tests and the Mann–Whitney U Test for continuous variables and chi-square or Fisher’s exact tests for categorical variables. Kaplan–Meier survival curves and log rank tests were used assess overall survival between groups. To ensure that censuring is non-informative, Kaplan–Meier survival curves and log rank tests were also used to compare shunt and transplant free survival. The significance level was set at 0.05 and SPSS version 24 (IBM Corp, Armonk, NY) was used for all analyses. Given the exploratory nature of this study and the concern for type 2 errors, we intentionally did not correct for multiple statistical tests and felt that each individual finding was important.

## Results

**Figure 1:**
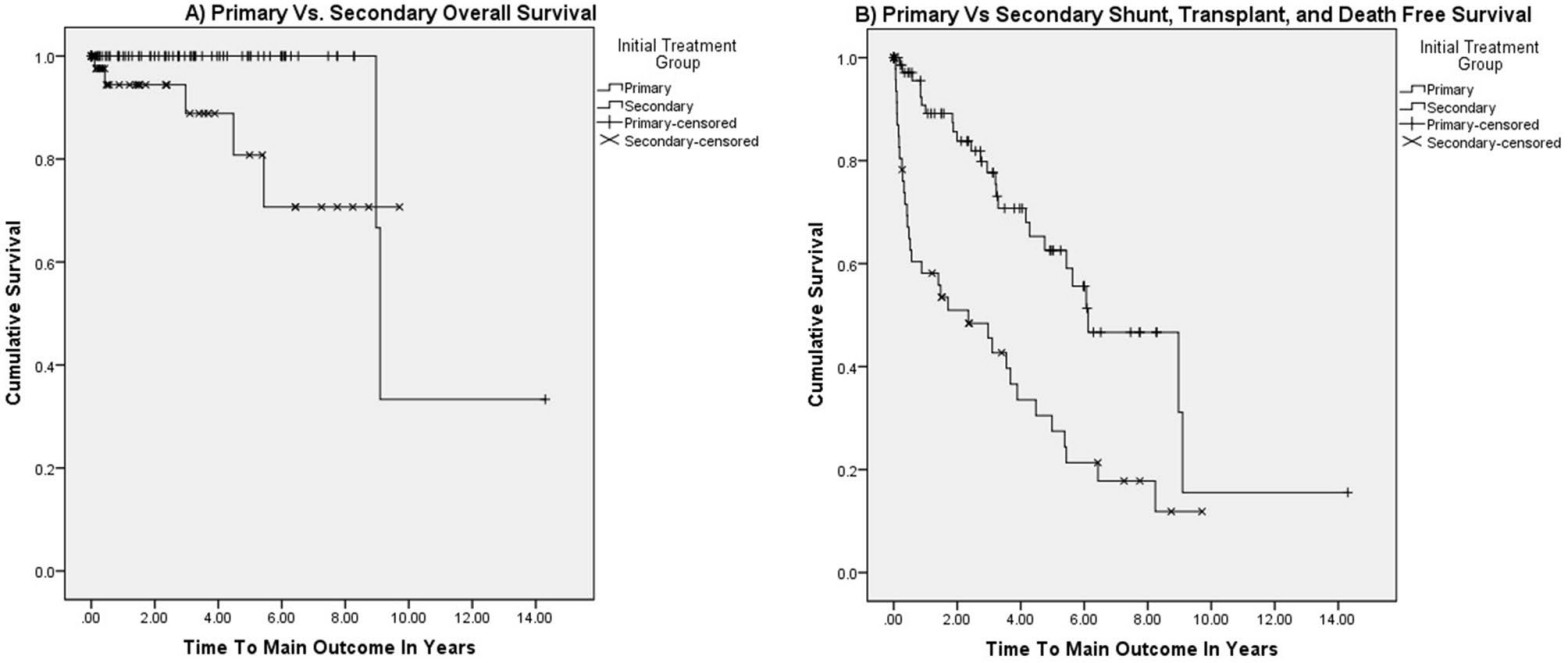
(**A**) Kaplan–Meier Overall Survival Curve for Primary and Secondary Endoscopic Prophylaxis (log-rank p = 0.023). (**B**) Kaplan–Meier Survival Curve without portosystemic shunt or liver transplant for Primary and Secondary Endoscopic Prophylaxis (log-rank p < 0.001).

Of patients who underwent endoscopy and had a diagnosis of PHTN, we identified 87 subjects who underwent primary prophylaxis endoscopy and 52 in the secondary prophylaxis group. Patient level characteristics are reported in Table 1. There were no significant differences in gender or racial/ethnic background. For primary etiology, patients with autoimmune hepatitis and cavernous transformation/thrombosis of the portal vein groups had significant differences between groups. Both groups presented at similar ages for their initial endoscopy. There was no significant difference in initial median PELD or MELD scores between both groups (Table 1). Median number of years of follow-up for all patients from first to last endoscopy was 2.13 years with a wide range (0–18.56 years, IQR 5.35 years) (Fig. 1A,B).

**Table 1:** Patient level characteristics of primary and secondary prophylactic endoscopy, p-value < 0.05 bolded.

	Primary prophylaxis		Secondary prophylaxis		p-value
	n	%	n	%	
Total patients	87	1	52	1	–
**Race/ethnicity**	–	–	–	–	–
American Indian/Alaskan native	1	11	0	0	0.626
Asian	3	3	1	2	0.520
African American	6	7	1	2	0.188
Caucasian	70	80	44	85	0.697
Hispanic/Latino	4	5	4	8	0.343
More than one race reported	2	2	0	0	0.390
Pacific Islander	0	0	1	2	0.374
Unknown/not documented	1	1	1	2	0.610
Female (%)	48	55	30	58	0.910
**Underlying diagnosis**	–	–	–	–	–
Alagille syndrome	5	6	1	2	0.270
Alpha-1-antitrypsin deficiency	5	6	2	4	0.475
Autoimmune hepatitis	8	9	0	0	**0.021**
Biliary atresia	14	16	16	31	0.068
Budd chiari	0	0	1	2	0.374
CTPV/PVT	10	11	14	27	**0.036**
Chronic heart disease	2	2	2	4	0.480
Congenital hepatic fibrosis	8	9	3	6	0.353
Cystic fibrosis related liver disease	13	15	4	8	0.160
Cystinosis	0	0	1	2	0.374
Glycogen storage disease	1	1	0	0	0.626
Joubert syndrome	1	1	0	0	0.626
Primary sclerosing cholangitis	5	6	1	2	0.270
VOD/SOS	1	1	0	0	0.626
Wilson's disease	2	2	0	0	0.390
Other (free text box)	12	14	7	13	1.000
Median age at first endoscopy (range), years	8.48 (0.56–17.87)	–	4.93 (0.50–17.88)	–	0.065
# of patients with a PELD score within 2 months of initial endoscopy (excluding CTPV and PVT)	46	53	21	40	–
Median PELD score (range)	0 (0–15.6)	–	0 (0–28.8)	–	**0.005**
# of patients with a MELD score within 2 months of initial endoscopy (excluding CTPV and PVT)	18	21	5	10	–
Median MELD score (range)	8 (6–26)	–	11 (9–17)	–	0.094
Median of total endoscopic procedures	3 (1–13)	–	6 (1–19)	–	**0.005**
Eradication of varices at final endoscopy	6	7	8	15	0.188
Number of patients who had bleeding despite primary prophylaxis	18	21	–	–	–
**Shunt**	5	6	18	35	** < 0.001**
Meso-rex shunt	0	0	4	8	–
Splenorenal shunt	5	6	13	25	–
Meso-caval shunt	0	0	1	2	–
Shunts who were eventually transplanted	1	1	2	4	–
Median number of endoscopies prior to shunt (range)	6 (1–10)	–	3 (0–12)	–	–
Liver transplants	21	24	14	27	0.082
Death	4	5	7	13	0.061
Was cause of death related to variceal complications or endoscopy	0	0	0	0	–

*CTPV* cavernous transformation of the portal vein, *PVT* portal vein thrombosis.

Endoscopic level characteristics can be found in Table 2. Indication for endoscopy was not included in statistical analysis as indications were part of the inclusion criteria for primary and secondary prophylaxis. However, if there was breakthrough GI bleeding, primary prophylaxis was associated with a lower number of hematemesis episodes (24% vs 51% of endoscopy for GI bleeding, p = 0.02). Primary prophylaxis was associated with significantly fewer admissions to the pediatric intensive care unit (PICU) at the time of endoscopy and lower graded esophageal varices. Additionally, secondary prophylaxis was associated with the patient having an active prescription for propranolol at the time of endoscopy (7% vs 15%, p < 0.001). There were no differences in the number of immediate and 2-week complications between groups. One patient treated with EVL for primary prophylaxis developed a partial esophageal stricture after 3 endoscopies. That patient required one-time balloon dilation without further management. There was a single stricture in the secondary prophylaxis group, again after 3 endoscopies, that did not allow the banding apparatus to pass preventing further prophylactic endoscopy. That patient was referred for portosystemic shunting, but their stricture remained asymptomatic. The remaining complications, such as chest pain and sore throat, were successfully managed with supportive care (Table 2).

**Table 2:** Endoscopy level characteristics of primary and secondary prophylactic endoscopy, p-value < 0.05 bolded.

	Primary prophylaxis		Secondary prophylaxis		p-value
	n	%	n	%	
Total number of endoscopy	339	–	326	–	–
**Indication for endoscopy**	–	–	–	–	–
Initial endoscopy for primary prophylaxis	76	22	0	0	–
Surveillance for primary prophylaxis	198	58	0	0	–
Management of active GI bleeding	29	9	90	28	–
Follow up after control of bleeding for eradication of varices	7	2	69	21	–
Surveillance for secondary prophylaxis	25	7	155	48	–
Anemia	3	1	4	1	–
Other	1	< 1	8	2	–
**If GI bleed, presenting symptom**	–	–	–	–	–
Hematemesis	7	24	46	51	**0.020**
Melena/bloody stools	16	55	30	33	0.060
Anemia	1	3	0	0	0.549
Hemoptysis	2	7	3	3	0.090
Other	3	10	11	12	1.000
**Admit location of endoscopy**	–	–	–	–	–
Same day surgery/out-patient	228	57	200	61	0.144
Floor	106	27	99	30	0.889
PICU	4	1	24	7	** < 0.001**
ED	0	0	1	< 1	0.489
Remaining is missing/unknown from EMR	1	< 1	2	< 1	–
**Variceal grade/size**	–	–	–	–	** < 0.001**
None	49	14	37	11	–
Grade 1/small/mild	128	38	83	25	–
Grade 2/medium/moderate	75	22	28	9	–
Grade 3/large/severe	56	17	115	35	–
Visualized, but not graded	31	9	63	19	–
Red wale sign	74	22	82	25	0.291
Bleeds with red wale sign	8	28	21	23	**0.017**
Gastric varices	74	22	108	33	**0.001**
Bleeds with gastric varices	3	10	29	32	** < 0.001**
Duodenal varices	9	3	2	< 1	0.080
Bleeds with duodenal varices	2	7	0	0	0.259
Portal hypertensive gastropathy	132	39	144	44	0.232
Bleeds with portal hypertensive gastropathy	12	41	39	43	** < 0.001**
**Treatment of varices**	–	–	–	–	–
None	201	69	134	46	** < 0.001**
Primary ligation	105	36	–	–	–
Secondary ligation	24	8	146	51	** < 0.001**
Primary sclerotherapy	0	0	–	–	–
Secondary sclerotherapy	1	< 1	32	11	** < 0.001**
Attempted but unsuccessful ligation	8	3	14	5	0.239
Propranolol usage at the time of endoscopy	23	7	50	15	** < 0.001**
Median NSBB dose, mg/kg/day (range)	0.79 (0.42–1.64)	–	1.06 (0.26–2.53)	–	0.118
How many bleeds occurred while on beta blocker	3	13	6	12	0.233
Octreotide used prior to endoscopy	7	2	15	5	0.099
**Complications, immediate**	4	1	8	2	0.173
Bleeding during procedure	1	< 1	6	2	–
Esophageal stricture	1	< 1	1	< 1	–
Desaturations/respiratory distress	2	< 1	1	< 1	–
**Complications within 2 weeks**	3	< 1	8	2	0.097
Recurrence of bleeding episode	0	0	1	< 1	–
Abdominal pain	1	< 1	2	< 1	–
Hemoptysis	0	0	1	< 1	–
Hypoglycemia episode following procedure	0	0	1	< 1	–
Chest pain	0	0	3	< 1	–
Febrile episode (also had chest pain)	0	0	1	< 1	–
Headache, fatigue, dizziness	1	< 1	0	0	–
Sore throat	1	< 1	0	0	–

**Figure 2:**
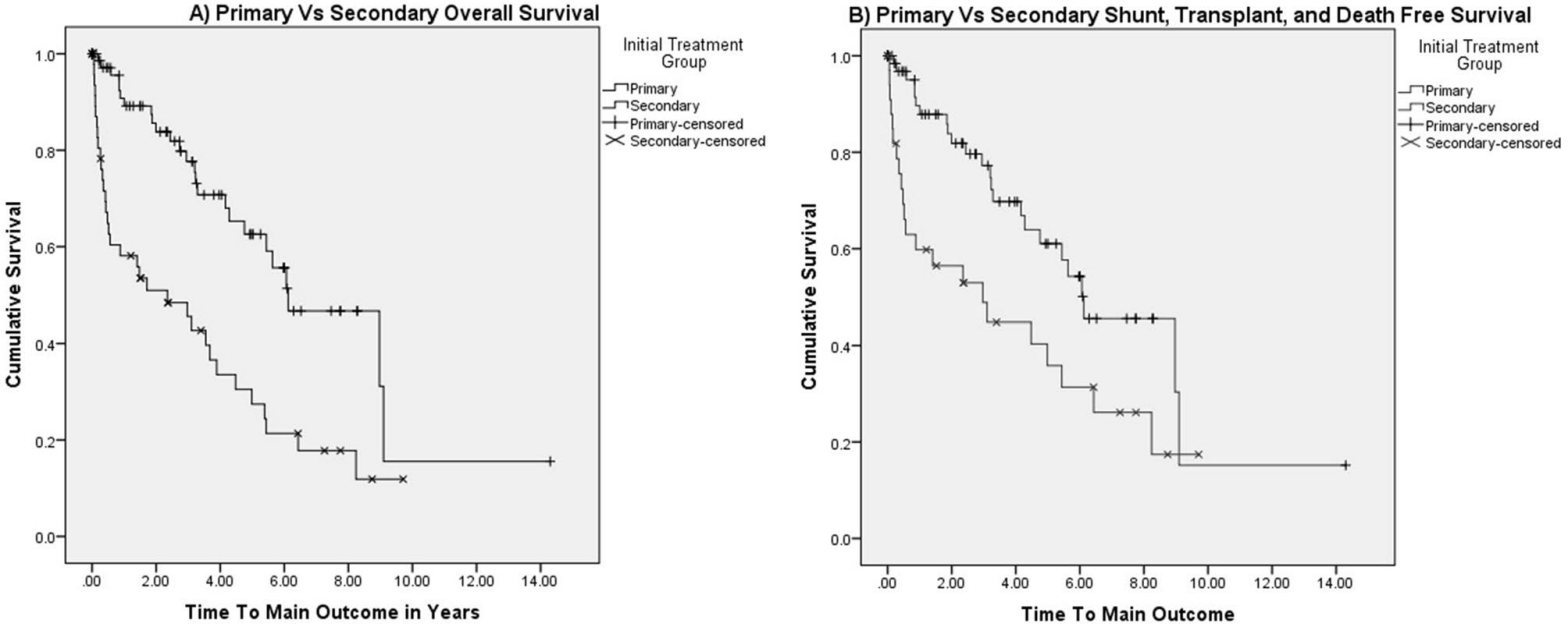
(**a**) Kaplan–Meier Overall Survival Curve for Primary and Secondary Endoscopic Prophylaxis excluding CTPV/PTV (log-rank p = 0.017). (**b**) Kaplan–Meier Survival Curve without portosystemic shunt or liver transplant for Primary and Secondary Endoscopic Prophylaxis excluding CTPV/PTV (log-rank p = 0.008).

Of note, patients who underwent primary prophylaxis had a lower median number of endoscopy throughout their treatment course (3 vs 6, p = 0.005, Table 1). Few patients in either group achieved complete eradication of varices at their final endoscopy without significant difference in either group (7% vs 15% of patients, p = 0.188); however, primary prophylaxis had a greater proportion of absent or Grade 1 varices at the time of endoscopy (Tables 1 and 2) and some patients were still actively undergoing therapy at the time this study was created. There were 18 (21%) patients in the primary prophylaxis group who eventually had an episode of gastrointestinal bleeding during their course. Patient characteristics of those who had breakthrough bleeding are summarized on Table 3. Interestingly, 7 (39%) of the patients with breakthrough bleeding had non-variceal causes of bleeding indentified at the time of endoscopy. However, these cases were still considered breakthrough bleeding as variceal bleeding could not be definitively ruled out. That said, patients in the primary prophylaxis group underwent fewer portosystemic shunts (6% vs 35% of patients, p < 0.001) with no significant differences in transplant (24% vs 27% of patients, p = 0.0819) or death (5% vs 13% of patients, p = 0.061) when analyzed via the Chi-square test of independence and Fisher’s exact test. We then analyzed outcomes with the Kaplan–Meier survival curves, which showed that patients in the primary prophylaxis group had better overall survival (Fig. 1A, log-rank p = 0.023) and survival without portosystemic shunt or LT (Fig. 1B, log-rank p < 0.001) than those in the secondary prophylaxis group. This pattern persisted when we repeated Kaplan–Meier analysis excluding patients with extrahepatic portal vein obstruction who did not have intrinsic liver disease (Fig. 2). There were too few patients in our group with extrahepatic portal vein obstruction alone to analyze and achieve meaningful or statistically significant results. Recorded deaths were not due to variceal bleeding or the endoscopic procedures themselves.

**Table 3:** Characteristics of those with breakthrough bleeding despite primary prophylaxis.

	n	%
Total patients	18	100
**Underlying diagnosis**	–	–
Alpha-1-antitrypsin deficiency	1	6
Autoimmune hepatitis	2	11
Biliary atresia	3	17
Cavernous transformation/thrombosis of the portal vein	1	6
Congenital hepatic fibrosis	1	6
Cystic fibrosis related liver disease	4	22
Primary sclerosing cholangitis	3	17
Other	3	17
Mean age at first endoscopy (SD)	8.39 (5.61)	–
Age range at first endoscopy	0.76–17.71	
**Variceal grade prior to breakthrough bleeding**	–	–
None	2	11
Grade 1/small/mild	10	56
Grade 2/medium/moderate	1	6
Grade 3/large/severe	5	28
Patient received ligation/sclerotherapy in endoscopy prior to breakthrough bleeding	3	17
**Presenting symptom**	–	–
Hematemesis	3	17
Melena/bloody stools	10	56
Anemia	1	6
Hemoptysis	1	6
Other	3	17
**Variceal grade at the time of breakthrough bleeding**	–	–
None	2	11
Grade 1/small/mild	6	33
Grade 2/medium/moderate	3	17
Grade 3/large/severe	6	33
Visualized but not graded	1	6
**Nonvariceal cause of bleeding identified**	7	39
Ulcer or gastritis	4	22
Anal fissure	1	6
Inflammatory bowel disease	1	6
*H. pylori*	1	6
**Treatment at the time of bleeding**	–	–
None	7	39
Secondary ligation	10	6
Secondary sclerotherapy	1	6
Mean years from first endoscopy to breakthrough bleeding (SD)	2.25 (2.28)	–
Range of years from first endoscopy to breakthrough bleeding	0.11–7.56	–
Mean years from previous endoscopy to breakthrough bleeding (SD)	0.70 (0.64)	–
Range of years from previous endoscopy to breakthrough bleeding	0.04–2.35	–
**Outcomes**	–	–
Portosystemic shunt	2	11
Liver transplant	6	33
Death	3	17
Shunt and transplant free survival	7	39

## Discussion

Gastroesophageal varices develop as the PSG rises to > 10 mmHg in adults and a PSG > 12 mmHg is predictive of those adults who will develop variceal hemorrhage, although pediatric confirmatory data are lacking and children may develop complications at lower gradients than adults^3,9,10^. The true incidence and prevalence of PHTN and variceal hemorrhage in pediatric patients is undereported. Current data are often limited to disease specific populations or registries. One study in long term survivors of biliary atresia without LT revealed up to two thirds of patients had PHTN and approximately 20% developed bleeding from esophageal varices^11^. The treatment of variceal hemorrhage is consistent across ages and includes pharmacologic measures (such as vasopressin or somatostatin) as well as endoscopic measures (such as EVL and sclerotherapy when EVL is not feasible). Refractory cases can be managed emergently with the Sengstaken-Blackmore tube. Shunt procedures (either transjugular intrahepatic portosystemic shunts or surgical) and transplantation are offered to the stabilized patient who has recurrent variceal hemorrhage not amenable to the above measures^3^.

Despite its routine use in adults, the primary prevention of gastrointestinal bleeding from varices remains underreported in pediatrics. Adult guidelines clearly recommend endoscopy in a patient with PHTN as the presence of varices can help determine prophylactic measures including NSBB and EVL^2^. These measures, have been shown to improve morbidity and mortality from variceal hemorrhage in adult patients with PHTN with no synergetic effect though and repeat endoscopy is recommended with intervals dependent on the progression of liver disease^12^.

Unfortunately, there is a paucity of data assessing the safety, efficacy, and outcomes of primary prophylaxis in pediatric PHTN; this includes studies on the use of both NSBB and endoscopy. As a result, there have been calls for further research acknowledging the difficulties of applying these results widely^4^. Guidelines, such as the Baveno VI Pediatric Satellite Symposium, have addressed this clinical question with a “do no harm“ approach until further research can be completed^13^. However, this recommendation requires discussion as institutions balance their patient populations with local expertise and available resources in an event that can have 6 week mortality as high as 8.8%^8^. While there are no published multi-centered, randomized, and prospective studies, there is a growing body of evidence that endoscopic primary prophylaxis may be safe and effective in pediatric portal hypertension.

In 2013, the French group Duché et al. reported their experience in primary and secondary prophylaxis in biliary atresia^6^. They found that primary prophylaxis was well tolerated and recommended continued surveillance even after eradication of varices in biliary atresia. This was then followed in 2017 by a retrospective study by Duché et al. for all causes of portal hypertension^14^. In this study they identified high risk varices (grade 3, grade 2 with red wale markings, and gastric varices) and noted that prevention of first bleed appeared to decrease morbidity and mortality. Additionally, Pimenta et al. found endoscopic primary prophylaxis to be superior to NSBB in a small study of 26 children with cirrhosis in its ability to reduce bleeding events^15^. Kang et al. found that EVL used to prevent rebleeding was safe and effective^16^. This is especially relevant as recent studies have shown that non-invasive measures have difficulty predicting gastrointestinal bleeding. Lampela et al. demonstrated that liver biochemistry labwork, liver stiffness, and predictive scores had difficulty predicting the presence of varices in pediatric patients with biliary atresia with similar conclusions by Angelico et al^17,18^. A recent study by Lee et al. compared primary and secondary endoscopic prophylaxis and found that primary prophylaxis was associated with less rebleeding and fewer endoscopic procedures; however, they did not find a difference in overall outcomes (alive with native liver) while our study suggests a difference in overall outcome^19^.

Pediatric guidelines provide a caveat that patients without easy access to appropriate levels of medical care may benefit from primary prophylaxis given the potential of rapid large volume hemorrhage^20^. Our practice historically established endoscopy for primary and secondary prophylaxis due to our significant rural patient population. This has led to a large pediatric population who have undergone both primary and secondary endoscopy prophylaxis with reportable clinical longitudinal data, outcomes, and complication rates to describe over a long period of time (Fig. 1A,B).

Importantly, we report few serious complications associated with prophylactic endoscopy (Table 2). The majority of complications resolved with supportive care and only 1 case required significant intervention (a single balloon dilation for a stricture). While 18 (21%) patients of the primary prophylaxis group had breakthrough bleeding, 7 (39%) of those cases had non-variceal causes of bleeding identified at the time of endoscopy and only 6 (33%) had large varices identified at the time of bleeding. These results suggest that prophylactic endoscopy in pediatric portal hypertension may be a safe alterantive to the watchful waiting approach.

There were significant differences and similarities noted between the primary and secondary prophylactic groups. However, it is important to note that our study was not designed to evaluate whether the differences were due to treatment strategy (primary vs second prophylaxis) or other confounding factors such as inherent patient characteristics. Neither group had significant differences in age of initial presentation or diagnoses other than autoimmune hepatitis and cavernous transformation of the portal vein/portal vein thrombosis (Table 1). Initial median PELD and MELD scores (excluding extrahepatic obstruction of the portal vein) did not show significant differences between the two groups. This suggests that subjects in the secondary prophylaxis group did not have significantly worse liver disease than those in the primary prophylaxis group. However, limitations to this interpretation include that PELD is not a prognostic indicator of variceal bleeding and the low numbers of available PELD and MELD scores in our patients that could skew results. Our primary prophylaxis group experienced a significantly decreased mean number of total endoscopies, usage of NSBB, PICU admissions, portosystemic shunts and higher rates of shunt and transplant free survival without significant complications. The improved shunt and transplant free survival also suggests decreased breakthrough bleeding as patient would be subsequently referred for shunting or transplant. Again, it remains unclear if this is due to treatment strategy or patient characteristics (such as severity of disease) and should be further studied.

Our study’s strength is longtidunal safety and efficacy over a long period of time that can add to the growing body of evidence that prophylactic endoscopy should be further studied in pediatric patients with portal hypertension to determine best practices. Limitations to this study include its retrospective nature and that patients were not randomized into either group. Importantly, there is also no control group who did not receive any prophylactic endoscopy. Our center does not a have large enough group of patients with portal hypertension who did not receive any prophylactic endoscopy to meaningfully study. While our analysis can describe safety and outcomes of pediatric prophylactic endoscopy, alone it is unable to fully address the debate between prophylactic endoscopy compared to watchful waiting. Additionally, our patient population reflects a wide etiology of PHTN and recommendations for specific diseases are not easily made. There may be disease specific factors that could effect outcomes. Comparing groups by specific disease resulted in too small of sample sizes to draw meaningful conclusions in our analyses. However, when we excluded extrahepatic portal vein obstruction alone from our Kaplan–Meier analysis, our findings persisted (Figs. 1 and 2). In Kaplan–Meier analysis, many patient were censored at an early stage of their follow up; however, log-rank analysis continued to show statistical significance. Lastly, our results also may better reflect our patient pool and local expertise rather than generalizable practice.

Further studies should be performed to clearly delineate morbidity and mortality between prophylactic endoscopy of either type and watchful waiting in the pediatric setting across a spectrum of disease. Ideally, these would be prospective and multicenter given the lower volumes of patients in pediatric centers compared to adult practices. Large scale multicentre patient registries can also contirubute further to our knowledge and understanding of PHTN and its management in children. However, this study provides supporting longitudinal evidence that prophylactic endoscopy can be considered a reasonably safe protocol to manage pediatric PHTN.

## Abbreviations

EVL: Endoscopic variceal ligation
LT: Liver transplant
MELD: Model for End-Stage Liver Disease
NSBB: Non-selective beta blockers
PELD: Pediatric End-Stage Liver Disease
PICU: Pediatric intensive care unit
PHTN: Portal hypertension
PSG: Portosystemic gradient
